# *TIM8* Deficiency in Yeast Induces Endoplasmic Reticulum Stress and Shortens the Chronological Lifespan

**DOI:** 10.3390/biom15020271

**Published:** 2025-02-12

**Authors:** Dong Tang, Wenbin Guan, Xiaodi Yang, Zhongqin Li, Wei Zhao, Xinguang Liu

**Affiliations:** 1 Guangdong Provincial Key Laboratory of Medical Immunology and Molecular Diagnostics, Institute of Aging Research, Guangdong Medical University, Dongguan 523808, China; td@gdmu.edu.cn; 2 School of Medical Technology, Guangdong Medical University, Dongguan 523808, China; gwb15625521878@163.com (W.G.); yxd0411@gdmu.edu.cn (X.Y.); lzql@gdmu.edu.cn (Z.L.)

**Keywords:** yeast, *TIM8*, ER stress, oxidative stress, CLS

## Abstract

Yeast *TIM8* was initially identified as a homolog of human TIMM8A/DDP1, which is associated with human deafness–dystonia syndrome. Tim8p is located in the mitochondrial intermembrane space and forms a hetero-oligomeric complex with Tim13p to facilitate protein transport through the TIM22 translocation system. Previous research has indicated that *TIM8* is not essential for yeast survival but does affect the import of Tim23p in the absence of the Tim8-Tim13 complex. Previous research on *TIM8* has focused mainly on its involvement in the mitochondrial protein transport pathway, and the precise biological function of *TIM8* remains incompletely understood. In this study, we provide the first report that yeast *TIM8* is associated with the endoplasmic reticulum (ER) stress response and chronological senescence. We found that deletion of *TIM8* leads to both oxidative stress and ER stress in yeast cells while increasing resistance to the ER stress inducer tunicamycin (TM), which is accompanied by an enhanced basic unfolded protein response (UPR). More importantly, *TIM8* deficiency can lead to a shortened chronological lifespan (CLS) but does not affect the replicative lifespan (RLS). Moreover, we found that improving the antioxidant capacity further increased TM resistance in the *tim8Δ* strain. Importantly, we provide evidence that the knockdown of TIMM8A in ARPE-19 human retinal pigment epithelium cells can also induce ER stress, suggesting the potential function of the *TIM8* gene in ER stress is conserved from budding yeast to higher eukaryotes. In summary, these results suggest novel roles for *TIM8* in maintaining ER homeostasis and CLS maintenance.

## 1. Introduction

The yeast gene *TIM8* encodes the mitochondrial intermembrane space protein Tim8, which is a component of the TIM22 complex. The TIM22 complex is a translocase on the mitochondrial inner membrane that mediates the import of target membrane proteins into the mitochondrial inner membrane [1].

The TIM22 translocation system includes five small Tim proteins, Tim8p, Tim9p, Tim10p, Tim12p, and Tim13p, and three membrane components, Tim18p, Tim22p, and Tim54p [1]. Tim8p binds to Tim13 to form a heterooligomeric complex and can be cross-linked to the mitochondrial inner membrane protein Tim23p [1,2]. Previous studies have reported that the Tim8-13 complex is not necessary for yeast survival but plays an important role in Tim23p import when the mitochondrial membrane potential is low [2]. *TIM8* and *TIM13* double mutants do not grow on yeast peptone dextrose (YPD) media at relatively low temperatures [1,3], and deletion of *TIM8* is synthetically lethal when in combination with temperature-sensitive *TIM10* mutation [4]. In *Trypanosoma brucei*, TbTim8/13, which is homologous to human Tim8 and Tim13, links to TbTim17 and is essential for optimal parasite proliferation [5].

*TIM8* has a human homolog gene termed TIMM8A. Previous studies have shown that mutation of the TIMM8A gene causes a neurodegenerative disorder called Mohr–Tranebjaerg syndrome (MTS), and the loss of TIMM8A can lead to abnormal mitochondrial morphology, mitochondrial dysfunction, and oxidative stress in cells [6]. However, there are no reports on whether *TIM8* or TIMM8A is involved in ER stress and the regulation of cellular senescence.

The ER is the main organelle involved in protein synthesis, folding, and processing in eukaryotic cells. Dysfunction of protein folding in the ER usually leads to the accumulation of misfolded and unfolded proteins within this organelle; this state is known as ER stress. ER stress can activate the UPR, which results in the upregulation of a cluster of genes involved in protein folding, quality control, and secretion to alleviate the accumulation of misfolded proteins in the ER [7,8]. Disturbances in protein homeostasis in the ER regularly accompany other types of cellular stress, such as oxidative stress, inflammation, and mitochondrial stress [9]. Moreover, studies have reported that ER stress is associated with cellular senescence and is involved in many age-associated neurodegenerative diseases [10,11].

In this study, we provide the first evidence that yeast *TIM8* may be involved in the ER stress response. *TIM8* deficiency in yeast leads to an enhancement in the basic UPR and increased resistance to the ER stress inducer TM, as well as a decreased CLS. Moreover, we show that the overexpression of *SOD2*, which is associated with decreased oxidative stress, in a *TIM8*-deficient strain could further improve yeast resistance to TM. More importantly, knockdown of the yeast *TIM8* human homolog TIMM8A in ARPE-19 human retinal pigment epithelium (RPE) cells also induces ER stress, suggesting the potential ER stress response function of the *TIM8* gene is conserved.

## 2. Materials and Methods

### 2.1. Strain Construction

All the *Saccharomyces cerevisiae* (*S. cerevisiae*) strains used in this study originated from the wild-type (WT) BY4742 strain and are listed in Table 1.

To construct the deletion strain *tim8Δ*, we generated a *TIM8* gene-specific disruption cassette containing the selectable marker *URA3* via polymerase chain reaction (PCR). The *URA3* marker was fused to the *TIM8* gene open reading frame (ORF) via homologous recombination [12]. The gene-specific disruption cassette was generated via PCR with the primers5′-AAGAGGTAAAAAGGAAAACAAATTTACAAACAACAAAGAAGATTGTACTGAGAGTGCAC-3′ and 5′-AAAGAGAATAATGACTCGGAGAGATAAATCGGTTTCATACTGTGCGGTATTTCACACCG-3′, and the purified PCR products were subsequently transformed into WT cells using lithium acetate (LiAc). The strain was subsequently grown in SD-URA media, and positive colonies were confirmed via PCR.

To construct the *SOD2* overexpression strains, we used the pAUR123 vector containing the *ADH1* promoter. The ORF of *SOD2* was amplified from the genome of BY4742 using the primers 5′-TATGGTACCATGTTCGCGAAAACAGCAGC-3′ with *KpnI* restriction sites and 5′-TATGAGCTCTCAGATCTTGCCAGCATCGA-3′ with *SacI* restriction sites and then cloned and inserted into pAUR123 to create the plasmid pAUR123*SOD2*. The recombinant plasmid was then transformed into the deficient mutant to generate the yeast strain *tim8ΔSOD2* with *SOD2* overexpression. The transformants were selected on YPD media plates supplemented with 0.2 g/mL aureobasidin A (AbA), and positive clones were verified via PCR.

The method used to make the *tim8ΔCTT1* OX and *tim8ΔHAC1* OX yeast strains was the same as the method used to make the *tim8ΔSOD2* yeast strain. The plasmid pAUR123*CCT1* was constructed previously in our laboratory [13]. The CDS of the spliced *HAC1* was amplified from the cDNA of a TM-treated WT yeast strain using the primers 5′-CGGGGTACCATGGAAATGACTGATTTTGAACT-3′ with *KpnI* restriction sites and 5′-CTAGTCTAGA TCATGAAGTGATGAAGAAATCA-3′ with *XbaI* restriction sites.

### 2.2. Spot Assay

Yeast strains were cultivated in YPD media and grown at 30 °C overnight. The second day, the strains were added to sterile water, and the absorbance of the suspension at 600 nm (OD_600_) was adjusted to 0.1, after which 5 µL of 5-fold serially diluted samples of the cell suspension were spotted onto YPD plates with or without TM. All the plates were incubated at 30 °C for two days, after which yeast growth was observed and photographed [14,15].

### 2.3. Growth Curve Assay

Growth curves were constructed from analysis with a Bioscreen C apparatus (Growth Curves, Helsinki, Finland). Briefly, a single isolated colony was inoculated into a cell culture tube filled with 3 mL of YPD medium and incubated overnight at 30 °C with continuous shaking at 150 rpm. The next day, the cultures were diluted with YPD medium to reach a final OD_600_ of 0.1, and the diluted yeast suspension was transferred to the wells of a Bioscreen plate. The inoculated plate was subsequently placed in the Bioscreen C instrument at 30 °C, and the OD_600_ was automatically measured every 2 h for more than 48 h [16,17]. Doubling time (Dt) and specific growth rate (µ) of the yeast cells were calculated as previously described [18]. Experiments were conducted at 30 °C, with three replicates per treatment.

### 2.4. Reactive Oxygen Species (ROS) Assay

Total intracellular ROS production was detected via dichlorodihydrofluorescein diacetate (DCFH-DA) staining. In brief, yeast cells were grown in YPD media with or without TM stress. Afterward, the cells were harvested, washed, resuspended in sterile phosphate-buffered saline (PBS) twice, and stained with 5 μM DCFH-DA at 30 °C in the dark for 1 h. The stained cells were detected via flow cytometry (BD FACSCanto II, USA) with excitation at 488 nm and emission at 525 nm [19,20]. For the detection of ROS in ARPE-19 cells, the cells were washed with PBS and incubated with 5 μM DCFH-DA at 37 °C for 30 min. Then, the cells were washed in PBS and trypsinized, and the fluorescence intensity in the yeast cells was measured via flow cytometry. The quantified data presented are from at least three independent experiments, and significant differences were determined via *t* tests. A *p* value less than 0.05 was considered to indicate statistical significance.

### 2.5. RLS Assay

This experiment was performed as previously described. Briefly, yeasts were aligned and cultured on YPD plates at 30 °C until buds were obtained. When the small buds completely grew, their mother cells were removed with a glass needle under an optical microscope and discarded, and the remaining daughter cells were named virgin mother cells. After virgin mother cell replication, the number of daughter cells was removed and recorded [21,22]. Statistical significance was calculated via the Wilcoxon rank sum test, and *p* < 0.05 was considered to indicate statistical significance.

### 2.6. CLS Assay

First, isolated yeast colonies were inoculated in 3 mL of synthetic complete liquid medium (SDC) and cultured overnight at 30 °C with shaking at 170 rpm. The next day, the cells were diluted to an OD_600_ of 0.1 in fresh SDC medium to a final volume of 15 mL, after which the cells were maintained at 30 °C with shaking for three days. After three days, the cultures were in the stationary phase, and proper dilutions of each sample were spotted onto YPD plates and incubated at 30 °C for 2 days, after which the colony-forming units (CFUs) were calculated. Spotting was repeated every three days until the end of the experiment, and the number of CFUs recorded on the first day was considered 100% survival [23,24].

### 2.7. Real-Time Polymerase Chain Reaction (RT-PCR)

Yeast strains were cultured in YPD medium to the exponential growth phase, and total RNA was collected for RT-PCR. Total RNA was extracted according to the RNA extraction kit standard protocol (Omega BioTek, USA). RT-PCR was performed with a LightCycler 480 instrument (Roche, USA) via the standard SYBR Green method. The number of transcripts for each target gene was normalized to that of the housekeeping gene *PRP8* [25,26]. The genes and sequences of primers used are listed in Table 2 and Table 3. The assay was repeated at least three times. Student’s *t* test was used for analysis, and a *p* value less than 0.05 was considered to indicate statistical significance.

### 2.8. Yeast HAC1 mRNA Splicing Pattern Assay

Total RNA and cDNA were obtained as described from the RT-PCR assay, and the cDNA was used as a template for detecting *HAC1* mRNA splicing via PCR. The primers with the *HAC1* intron used for PCR were 5′-CCGTAGACAACAACAATTTG-3′ and 5′-CATGAAGTGATGAAGAAATC-3′. The PCR products were resolved via electrophoresis on a 1.5% agarose gel, and the sizes of the amplified products, 433 bp and 181 bp, were observed. The image was inverted for clarity. The results were analyzed using ImageJ V1.8.0 software (NIH, Bethesda, MD, USA) [27].

### 2.9. Mitochondrial Function Assay

2,3,5-Triphenyltetrazolium (TTC) was used to determine yeast mitochondrial function. Briefly, yeast cells were spread on YPD agar media and cultured for 48 h in a 30 °C incubator until colonies formed, after which the plates were completely overlaid with TTC agar (1.5% low-melting point agarose and 0.1% TTC). The numbers of red and white colonies were recorded and analyzed, and the results are given as the ratio of white colonies [28].

### 2.10. Cell Culture

ARPE19 human retinal epithelial cells were obtained from iCell Bioscience, Inc. (Shanghai, China). The cells were cultured in Dulbecco’s modified Eagle’s medium/nutrient mixture F-12 (DMEM/F12) (Gibco, USA) supplemented with 10% fetal calf serum in a 5% CO2/95% air (*v*/*v*) incubator at 37 °C.

### 2.11. Small Interfering RNA (siRNA) Transfection

TIMM8A knockdown was conducted via transfection of specific siRNAs using Lipofectamine RNAiMAX (Thermo Fisher Scientific, USA) according to the manufacturer’s instructions. siRNAs (genOFFTM st-h-TIMM8A_001 and genOFFTM st-h-TIMM8A_002) targeting human TIMM8A mRNA were designed and synthesized by Ribobio (Guangzhou, China). For each transfection, 50 nM siRNA was used.

### 2.12. Western Blot Analysis

Proteins from whole cells were extracted using RIPA lysis buffer. The total protein extracted was analyzed via sodium dodecyl sulfate-polyacrylamide gel electrophoresis (SDS-PAGE) and transferred to a polyvinylidene fluoride (PVDF) membrane (Millipore, USA). The membrane was blocked with 3% bovine serum albumin (BSA) in Tris-buffered saline containing Tween 20 (TBST; 20 mM Tris-HCl pH 7.5, 150 mM NaCl, 0.1% Tween 20) before incubation with the primary antibody at 4 °C overnight followed by incubation with an alkaline phosphatase (AP)-labeled secondary antibody. The bands were visualized with nitro blue tetrazolium/4-bromo-5-chloro-indolyl phosphate (NBT/BCIP) reagent (Thermo Fisher Scientific, USA). The antibodies used were as follows: anti-GAPDH (1:1000, sc-166545, Santa Cruz Biotechnology, USA), anti-XBP1s (1:1000, 40435T, Cell Signaling Technology, USA), anti-GFRP78 (1:1000, 11587-1-AP, Proteintech, China), and anti-TIMM8A (1:1000, 1 11179-1-AP, Proteintech, China).

### 2.13. Measurement of the Intracellular Calcium Content

The intracellular calcium content was measured with Fluo-3 AM and flow cytometry [29,30]. In brief, the cells were incubated in HBSS containing 3 μM Fluo-3 for 30 min at 37 °C in the dark. The cells were then washed in PBS and trypsinized, and the fluorescence intensity was measured via flow cytometry with excitation at 506 nm and emission at 526 nm. The quantified data from at least three independent experiments are presented, and significant differences between the results were determined via *t* tests. A *p* value less than 0.05 was considered to indicate statistical significance.

## 3. Results

### 3.1. TIM8 Deficiency Increases Yeast Resistance to the ER Stress Inducer TM

**Figure 1:**
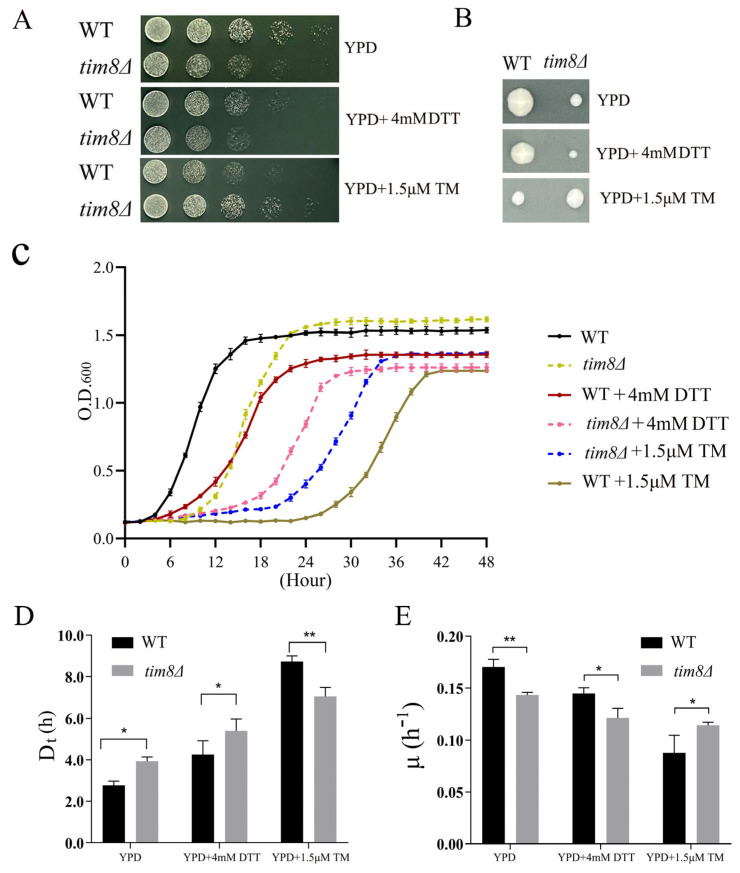
*tim8Δ* cells displayed increased resistance to TM. (**A**) The WT and *tim8Δ* strains were serially diluted and spotted on YPD plates containing 1.5 μM TM or 4 mM DTT. The plates were incubated at 30 °C until colonies formed, after which photographs were taken. (**B**) Colony-forming unit assays. (**C**) Growth curves of the WT and *tim8Δ* strains with or without TM/DTT were constructed after automatic measurements were taken every 2 h for more than 48 h. (**D**) Doubling time (Dt) and (**E**) specific growth rate (µ) assays of the yeast cells, data are presented as the mean ± standard deviation, statistical analyses were performed using a two-way ANOVA test. * indicates *p* < 0.05; ** indicates *p* < 0.01.

To investigate the potential involvement of *TIM8* in the yeast ER stress response, we first constructed a *tim8Δ* mutant strain derived from the WT BY4742 strain. Both the *tim8Δ* and WT strains were spotted onto YPD media with or without the ER stress inducer TM/DTT. The results revealed that the growth ability of *tim8Δ* cells was clearly suppressed compared with that of the WT cells under unstressed conditions, whereas surprisingly, the growth ability of *tim8Δ* cells clearly increased compared with that of the WT yeast cells in the presence of 1.5 μM TM (Figure 1A). This result was confirmed by the colony-forming unit assay (Figure 1B) and growth curve assay results (*tim8Δ* cells grow faster and enter exponential growth phase earlier than WT yeast cells under the TM stressed condition) (Figure 1C). Moreover, the doubling time assay (Figure 1D) and specific growth rate assay (Figure 1E) also showed that the growth ability of *tim8Δ* cells clearly increased compared with that of the WT yeast cells in the presence of TM. Interestingly, *tim8Δ* cells exhibited the same growth inhibition (compared with WT cells) in the presence of the other ER stress inducer, DTT.

### 3.2. TIM8 Deficiency Leads to Both Oxidative Stress and ER Stress in Yeast Cells

**Figure 2:**
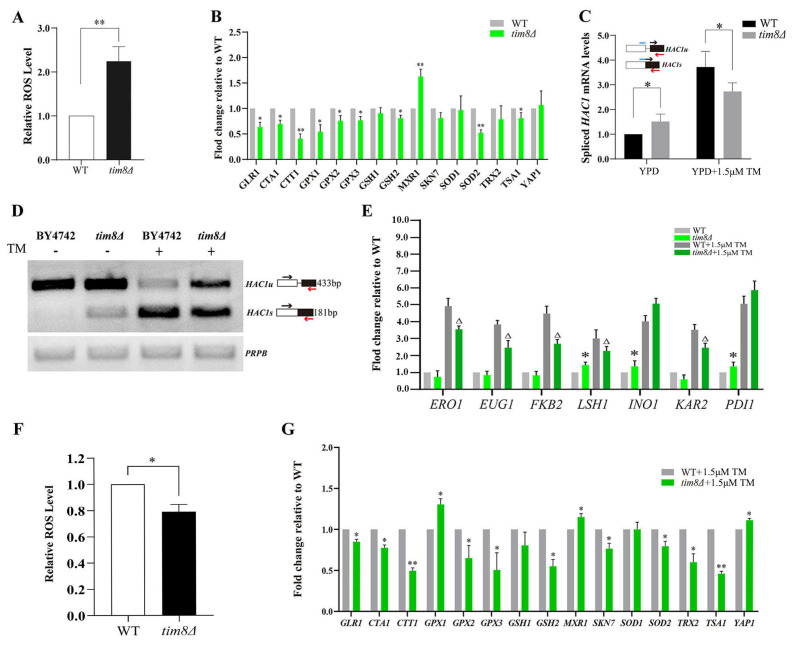
*TIM8* deficiency leads to an imbalance in the antioxidant system in yeast. (**A**) The relative ROS levels in *tim8Δ* cells were significantly increased under normal conditions. ** indicates *p* < 0.01, the control’s error was propagated into the normalized values. (**B**) mRNA expression levels of oxidative stress-related genes in the WT and *TIM8*-deficient strains under unstressed conditions measured via RT-PCR. The number of transcripts was normalized to that of *PRP8*. * indicates *p* < 0.05; ** indicates *p* < 0.01, the control’s error was propagated into the normalized values. (**C**) The expression levels of spliced *HAC1* mRNA (*HAC1s*) were detected by RT–PCR. The number of transcripts was normalized to that of the housekeeping gene *PRP8*. * indicates *p* < 0.05, the control’s error was propagated into the normalized values. (**D**) *HAC1* mRNA splicing pattern in *TIM8*-deficient and WT strains detected via agarose gel electrophoresis. *HAC1s*, spliced *HAC1* mRNA. *HAC1u*, unspliced *HAC1* mRNA. *PRP8* was used as the control. The image was inverted for clarity. The intensity of each band was quantified densitometrically using ImageJ software V1.8.0. (**E**) The mRNA levels of UPR genes in the WT strains and *TIM8*-deficient strains measured via RT-PCR under stressed (1.5 μM TM) and unstressed conditions, and the expression was normalized to that of the housekeeping gene *PRP8*. All data represent the mean ± S.D. of three biological replicates, with * (*p* < 0.05) indicating unstressed *tim8Δ* vs. unstressed WT, and Δ (*p* < 0.05) indicating TM-stressed *tim8Δ* vs. TM-stressed WT, the control’s error was propagated into the normalized values. (**F**) The relative ROS levels in *tim8Δ* cells significantly decreased under 1.5 μM TM stress, and the control’s error was propagated into the normalized values. * indicates *p* < 0.05. (**G**) mRNA expression levels of oxidative stress-related genes in the WT and *tim8Δ* strains under 1.5 mM TM stress. * indicates *p* < 0.05; ** indicates *p* < 0.01, the control’s error was propagated into the normalized values. Original images of (**D**) can be found in Supplementary Materials (File S1).

Previous studies have reported that the depletion of human TIMM8A (homolog of yeast *TIM8*) in HEK293 and SH-SY5Y cells results in cellular oxidative stress and sensitivity to oxidative stress-mediated apoptosis [6]. We thus wondered whether the deletion of *TIM8* in yeast has the same effect. First, we found that the intracellular ROS level was increased in *tim8Δ* cells (Figure 2A). We further measured the mRNA expression levels of several oxidative stress-related genes, and the results revealed that the expression of most of the genes investigated was reduced in the *tim8Δ* strain (Figure 2B).

Furthermore, we examined the growth ability of *tim8Δ* and WT cells on plates containing different oxidants, such as tert-butyl hydroperoxide (TBHP), cumene hydroperoxide (CHP), and H_2_O_2_. The growth ability of the *tim8Δ* strain was enhanced in YPD medium supplemented with TBHP, whereas under CHP and H_2_O_2_ stress, the growth inhibition (compared with that of the WT strain) of the *tim8Δ* strain was the same as that under unstressed conditions (File S4).

Oxidative stress and ER stress are closely interconnected biological processes [31]. Therefore, we next analyzed the splicing pattern of *HAC1* mRNA, a marker of ER stress in yeast cells [32]. Both RT-PCR and semiquantitative PCR revealed that spliced *HAC1* mRNA levels increased in *tim8Δ* cells under unstressed conditions compared with those in WT yeast cells, suggesting that *TIM8* deletion induced ER stress under normal growth conditions. In contrast, the spliced *HAC1* mRNA levels were lower in *tim8Δ* cells than in WT yeast cells after treatment with TM (Figure 2C,D), indicating that the ER stress was not as severe in *tim8Δ* cells as in the WT yeast cells.

In addition, we detected the transcription patterns of the canonical UPR target genes in the *tim8Δ* strain in the presence and absence of TM stress. Compared with those of the WT strain, the transcription levels of *LHS1*, INO1, and *PDI1* were increased under unstressed conditions, while the transcription levels of ERO1, EUG1, *FKB2,* and *KAR2* were not different (Figure 2E). However, the expression of almost all UPR genes decreased in the *tim8Δ* strain, except for two, *INO1* and *PDI1*, under TM-stressed conditions.

Moreover, we found that ROS production was lower in *tim8Δ* cells than in WT yeast cells under TM stress (Figure 2F), and interestingly, the expression levels of most of the investigated antioxidant genes were also lower in *tim8Δ* cells under TM stress (Figure 2G).

### 3.3. TIM8 Deficiency Leads to a Shortened CLS

**Figure 3:**
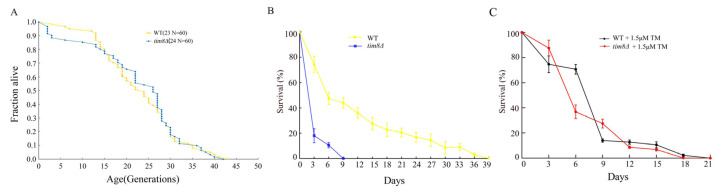
The RLS and CLS of *tim8Δ* cells. (**A**) The RLS of the WT and *tim8Δ* strains (the mean lifespans are shown in parentheses; N indicates the total number of mother cells). *TIM8* deletion did not result in significant differences in the RLS. The experiments were performed in duplicate, and the differences were analyzed for statistical significance via the Wilcoxon rank sum test. (**B**) The CLS of the WT and *tim8Δ* strains was determined in synthetic complete liquid medium under the unstressed condition. (**C**) The CLS of the WT and *tim8Δ* strains under the TM-stressed condition.

Oxidative stress and ER stress are closely related to the aging process in yeast, so we examined the lifespan of the *tim8Δ* strain. Aging in yeast is assayed primarily by measuring RLS (defined as the number of total daughter cells produced by an individual mother cell) or CLS (defined as the length of time a population of yeast cells remains viable in the stationary phase) [33]. We found that the mean RLS of the *TIM8* deletion strain was 24 generations, while the mean RLS of the WT strain was 23 generations, and the difference was not significant (Figure 3A). On the other hand, the CLS assay revealed that the maximum lifespan of the *tim8Δ* strain was less than 9 days, whereas the maximum lifespan of the WT strain was more than 30 days (Figure 3B).

We also quantified the CLS for both *tim8Δ* and WT strains in the presence of TM (Figure 3C). The results showed that, when stressed with TM, the CLS of the *tim8Δ* cells was increased (similar to the stressed WT strain cells) compared with that of the unstressed *tim8Δ* cells, while the CLS of the WT cells was decreased compared with that of the unstressed WT cells.

### 3.4. TIM8 Deficiency Impaired the Mitochondrial Respiration Capacity of Yeast

**Figure 4:**
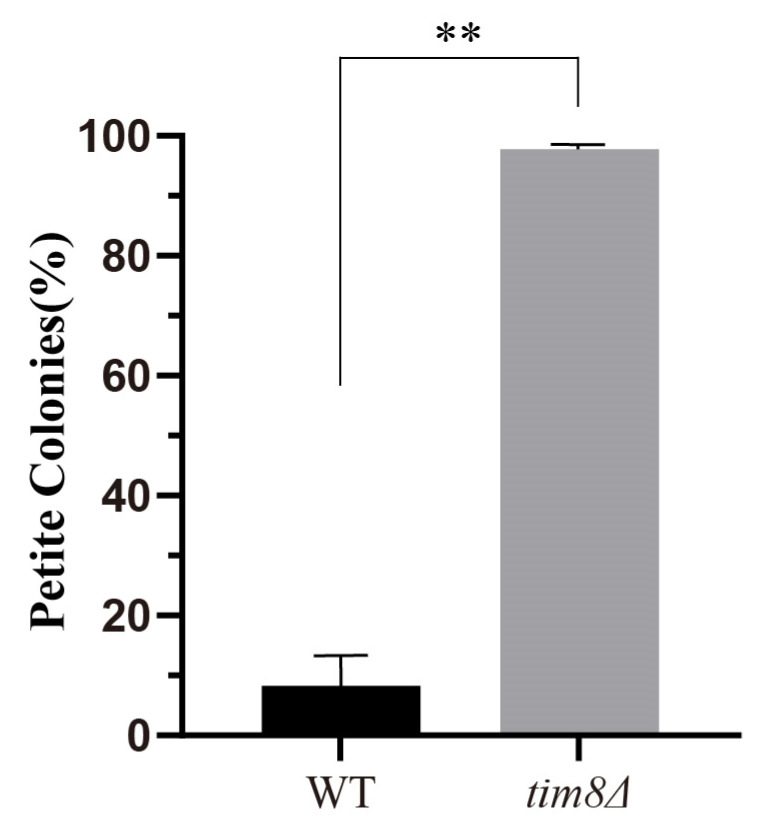
The mitochondrial respiration capacity decreased in *tim8Δ* cells. Compared with WT cells, *tim8Δ* cells formed more petite colonies. Statistical significance was analyzed by χ^2^-test, and a *p* value less than 0.05 was considered to indicate statistical significance. ** *p* < 0.01.

Previous studies have shown that the depletion of the homolog of yeast *TIM8* in mammalian cells leads to mitochondrial dysfunction [6]. Thus, we also investigated yeast mitochondrial respiration capacity via TTC overlay, and the results revealed that the percentage of respiration-deficient petite *tim8Δ* cells increased compared with the WT, which suggested that *tim8Δ* cells had an impaired mitochondrial respiration capacity (Figure 4).

### 3.5. SOD2 Overexpression Enhances TM Resistance in the tim8Δ Strain

**Figure 5:**
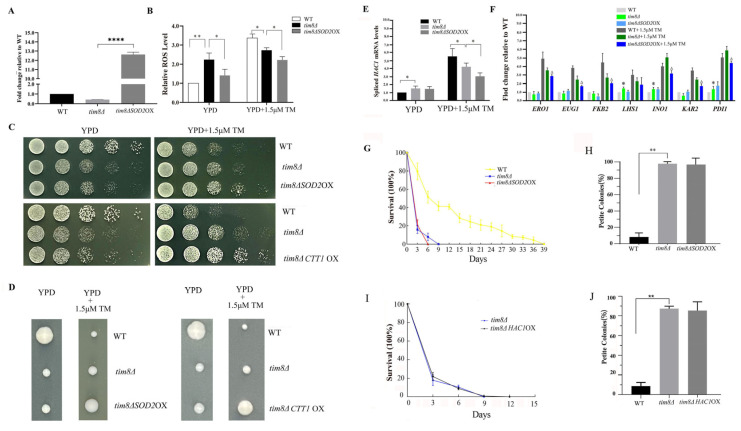
*SOD2* overexpression enhances TM resistance in the *tim8Δ* strain. (**A**) The *SOD2* gene was highly expressed in the overexpression strains. Statistical significance was analyzed by Student’s *t* test, and a *p* value less than 0.05 was considered to indicate statistical significance, **** *p* <0.0001 the control’s error was propagated into the normalized values. (**B**) Overexpression of *SOD2* reduced ROS with or without TM stress. Statistical significance was analyzed by Student’s *t* test, and a *p* value less than 0.05 was considered to indicate statistical significance. * *p* < 0.05, ** *p* <0.01, the control’s error was propagated into the normalized values. (**C**) The WT, *tim8Δ*, *tim8Δ SOD2*OX, and *tim8Δ CCT1*OX strains were grown on YPD plates with or without 1.5 μM TM. (**D**) Colony-forming unit assays. (**E**) mRNA expression levels of spliced *HAC1*. Statistical significance was analyzed by Student’s *t* test, and a *p* value less than 0.05 was considered to indicate statistical significance. * *p* < 0.05, the control’s error was propagated into the normalized values. (**F**) mRNA levels of UPR genes in the WT, *tim8Δ,* and *tim8Δ SOD2* OX strains were measured via RT-PCR under stressed (1.5 μM TM) and unstressed conditions. The results were analyzed by Student’s *t* test. All data represent the mean ± S.D. of three biological replicates, with * (*p* < 0.05) indicating unstressed *tim8Δ* vs. unstressed WT, and Δ (*p* < 0.05) indicating TM-stressed *tim8Δ* vs. TM-stressed WT, the control’s error was propagated into the normalized values. (**G**) CLS of the WT, *tim8Δ,* and *tim8Δ SOD2* OX strains. (**H**) Overexpression of *SOD2* was unable to restore respiration capacity in the *tim8Δ* strain. The results were analyzed by χ2-test, and a *p* value less than 0.05 was considered to indicate statistical significance. ** *p* < 0.01. (**I**) CLS of the *tim8Δ* and *tim8Δ HAC1* OX strains. (**J**) Overexpression of *HAC1* was unable to restore respiration capacity in the *tim8Δ* strain. Statistical significance was analyzed by χ^2^-test, and a *p* value less than 0.05 was considered to indicate statistical significance. ** *p* < 0.01.

In the previous section, we showed that the expression level of the mitochondrial Mn superoxide dismutase-encoding gene *SOD2* was decreased under both unstressed and TM-stressed conditions; most importantly, the expression level of the *SOD2* gene was most significantly reduced under unstressed conditions (Figure 2B). Therefore, we overexpressed the *SOD2* gene in the *tim8Δ* strain (Figure 5A) and found that the ROS levels were lower than those in the WT strain in both the presence and absence of TM stress (Figure 5B).

The spot assay and colony-forming unit assay revealed that *SOD2* overexpression enhanced the growth ability of the *tim8Δ* strain on agar plates containing TM (the improvement was not obvious under normal conditions). We also overexpressed another antioxidant gene in *tim8Δ* cells, *CTT1* (File S3), which encodes a peroxisomal catalase that can break down H_2_O_2_, and the effect was similar to that of *SOD2* overexpression (Figure 5C,D).

RT-PCR revealed that the expression level of spliced *HAC1* in *tim8Δ SOD2*OX cells was noticeably decreased upon TM treatment but was not significantly different from that of the WT strain under unstressed conditions (Figure 5E); moreover, the UPR gene expression profile was in accordance with the expression pattern of spliced *HAC1* (Figure 5F).

The lifespan assay revealed that the CLS of the *tim8ΔSOD2*OX strain did not differ from that of the *TIM8* deletion strain (Figure 5G). Additionally, there was no significant difference in the TTC assay results between the *tim8ΔSOD2* OX and *tim8Δ* strains (Figure 5H).

Moreover, we overexpressed spliced *HAC1* in *tim8Δ* to test whether upregulating the ER stress response could restore CLS in *tim8Δ* (File S3). The results showed that the *tim8Δ HAC1*OX cells exhibited a CLS similar to that of *tim8Δ* cells (Figure 5I). In addition, the TTC assay results also showed there was no difference between the *tim8Δ HAC1* OX strain and the *tim8Δ* strains (Figure 5J).

### 3.6. Knockdown of TIMM8A Induces ER Stress in ARPE-19 Cells

**Figure 6:**
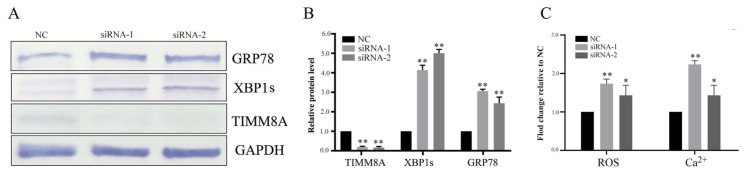
Knockdown of TIMM8A induces ER stress in ARPE-19 cells. (**A**) siRNA was transfected into ARPE-19 cells to silence TIMM8A gene expression. The cell lysates were analyzed via Western blotting with antibodies specific for GRP7, XBP1s, and TIMM8A. The changes in protein expression were normalized to that of GAPDH. (**B**) Quantification of the relative TIMM8A, GRP78, and XBP1s protein levels. Statistical significance was analyzed by Student’s *t* test, and the control’s error was propagated into the normalized values. ** *p* <0.01, the control’s error was propagated into the normalized values. (**C**) Relative ROS and intracellular calcium levels in siRNA-transfected ARPE-19 cells. Statistical significance was analyzed by Student’s *t* test, and the control’s error was propagated into the normalized values. * *p* < 0.05, ** *p* <0.01, the control’s error was propagated into the normalized values. Original images of (**A**) can be found in Supplementary Materials (File S2).

We next knocked down the TIMM8A gene in ARPE-19 human RPE cells via RNA interference and found that the protein levels of GRP78 and spliced XBP1 (XBP1s), two ER stress markers, notably increased, suggesting that knockdown of TIMM8A, the homologous human gene of yeast TIM8, also induces ER stress. Moreover, we also found that the knockdown of TIMM8A in ARPE-19 cells can increase both the ROS and intracellular calcium levels (Figure 6).

## 4. Discussion

### 4.1. TIM8 Deficiency Induces ER Stress Response

The yeast gene *TIM8* was initially identified as a homolog of human TIMM8A/DDP1, and mutation of the latter gene is associated with human deafness–dystonia syndrome [4,34]. Previous studies of *TIM8* have mainly focused on its involvement in the TIM22 protein import pathway, which mediates the import of membrane proteins into the mitochondrial inner membrane [1], but the precise biological function of *TIM8* remains largely unknown.

In this study, we first demonstrated that *TIM8* deficiency could induce the ER stress response. We found that the growth ability of *tim8Δ* cells was clearly suppressed compared with that of WT cells under normal physiological conditions. Upon applying TM stress, the growth ability of *tim8Δ* cells clearly increased compared with that of the WT yeast cells (Figure 1A–E).

ER stress can activate the UPR, resulting in the upregulation of a cluster of genes involved in protein folding, quality control, and secretion to restore ER homeostasis [7,8]. Unlike the three UPR pathways that exist in mammalian cells, only one UPR pathway, which is mediated by IRE1, has been reported in budding yeast [35]. The accumulation of misfolded and unfolded proteins in the ER leads to activation of the ER stress sensor Ire1p, which excises the translation inhibitory intron of *HAC1* mRNA. This induces synthesis of the transcription factor Hac1p and the subsequent Hac1p-mediated upregulation of a cluster of genes involved in protein folding, quality control, and secretion to ultimately reduce the accumulation of misfolded and unfolded proteins in the ER [36].

We showed that the levels of UPR activity marker, the spliced *HAC1* mRNA, were increased in *tim8Δ* cells under unstressed conditions, and accordingly, three UPR target genes were notably upregulated in *tim8Δ* cells (Figure 2C–E). These results indicate that basic UPR activity and ER stress are increased in *tim8Δ* cells. It has been suggested that moderate ER stress is beneficial for cell growth but that persistent or chronic ER stress can trigger cell death [11,37]. We speculated that the increase in ER stress might explain the growth inhibition of *tim8Δ* cells under normal conditions. On the other hand, we found that the intracellular ROS level increased, and the expression levels of most of the investigated antioxidant genes were reduced in *tim8Δ* cells under normal conditions, suggesting the occurrence of oxidative stress (Figure 2A,B). This would be another reason for *tim8Δ* cell growth inhibition under unstressed conditions.

However, why does *TIM8* deficiency induce ER stress? Research has suggested that there is crosstalk between factors involved in ER stress and oxidative stress. On the one hand, oxidative stress can disrupt redox homeostasis in the ER, leading to improper disulfide bond formation and the accumulation of misfolded proteins; on the other hand, misfolded proteins trigger the excessive accumulation of intracellular ROS [38,39,40]. Given these phenomena, we presume that increased oxidative stress might be the cause of the observed ER stress in *tim8Δ* cells. Notably, ER stress and oxidative stress can reciprocally induce each other and may be coregulated via a positive feedback loop. Given that both oxidative stress and ER stress were increased in *tim8Δ* cells under normal conditions, we cannot speculate which type of stress is the first stress to occur in these cells.

More importantly, we should note that the increased basal ER stress in *tim8Δ* mutants might result from primary stress (oxidative stress or other stresses) due to the absence of *TIM8* rather than an exacerbated stress because of the downregulation of the ER stress response pathway.

The second question is why was the growth ability of *tim8Δ* cells clearly increased and better than that of WT cells under TM-stressed conditions? We evaluated the UPR activity in *tim8Δ* cells and WT cells under TM-stressed conditions and found that the spliced *HAC1* mRNA levels were decreased in *tim8Δ* cells compared with those in WT yeast cells. Accordingly, the expression of almost all of the investigated UPR genes decreased in the *tim8Δ* strain (Figure 2C–E). Interestingly, the ROS level was also lower in *tim8Δ* cells than in WT yeast cells under TM stress (Figure 2F). Taken together, these observations implied that there was mild ER stress in *tim8Δ* cells compared with WT yeast cells in the presence of TM.

TM is a common compound that can induce ER stress by inhibiting the protein N-glycosylation pathway [41], which leads to the accumulation of unfolded or misfolded proteins in the ER and thereby causes ER stress. We also found the *tim8Δ* cells exhibited the same growth inhibition (compared with the WT cells) when another ER stress inducer, DTT, was applied, which disrupted the formation of disulfide bonds and led to the accumulation of unfolded proteins in the ER [42]. Thus, we speculate that the activity of the protein N-glycosylation pathway might be enhanced in *tim8Δ* cells.

Interestingly, two large-scale analyses to identify yeast mutants that are either sensitive or resistant to particular compounds have reported that *TIM13* (another member of the Tim8-Tim13 complex) deficiency cells (*tim13Δ*) were resistant to 0.6 μM TM [43] and sensitive to the genotoxic reagent methyl methanesulfonate (MMS, could result in DNA damage and induce a ROS stress response in budding yeast) [44,45]. These reports suggested that *TIM13* may also play a role in ER stress and oxidative stress.

### 4.2. tim8Δ Cells Exhibit a Shortened CLS

An increasing number of studies have revealed that both ER stress and oxidative stress are associated with the cellular senescence process [11,27,46]. According to the free radical theory, oxidative stress caused by excessive intracellular ROS is a major contributor to aging in yeast and mammalian cells [47]. For example, deletion of the *PEP4* gene in yeast led to oxidative stress and increased sensitivity to hydrogen peroxide, as well as a shortened CLS [48]. With respect to ER stress, moderate ER stress triggers an adaptive UPR that is beneficial for cellular survival, whereas persistent or acute ER stress and the UPR accelerate the apoptotic process and lead to cell death. For example, *GAS1*-deficient yeast cells presented increased intracellular UPR activity and shortened RLS [15].

Interestingly, we found that the CLS of *tim8Δ* cells was significantly lower than that of WT strain cells, whereas the RLS of *tim8Δ* cells was similar to that of WT strain cells (Figure 3A,B).

Many previous studies have shown that both environmental and genetic factors can affect replicative aging and chronological aging and have suggested that some molecular factors are correlated and likely play a causal role in determining both CLS and RLS, whereas some causes of aging appear to be private for each type of yeast aging [48,49]. For example, deletion of yeast *SIR2* could increase rDNA instability and dramatically shorten the RLS but does not affect the CLS in standard media [33].

We do not know why *TIM8* deficiency affects only the CLS. It has been reported that mitochondrial function, more specifically respiration, severely affects the CLS of yeast cells [50,51]. Specifically, the CLS of budding yeast is very sensitive to medium acidification. Under standard glucose culture conditions (medium containing 2% glucose), cells initially ferment the glucose to ethanol. After glucose depletion, the ethanol is metabolized, leading to the production of acetic acid, which is toxic to yeast cells and induces chronological senescence. During this process, yeast cells with abnormal mitochondrial function may produce more metabolic acid and further reduce the CLS [52,53]. Based on these findings, the observed abnormal mitochondrial respiration capacity may be another explanation for the decreased CLS of *tim8Δ* cells.

Moreover, in the previous section, the growth assay suggested a protective effect of *tim8Δ* against TM. However, it is unclear whether the observed growth rate improvement was due to reduced cell death or faster division. Given this, we further assessed whether *TIM8* deficiency also protected against TM in a CLS context. The results showed that, when stressed with TM, the CLS of the *tim8Δ* cells was increased (similar to the stressed WT strain cells) compared with that of the unstressed *tim8Δ* cells, while the CLS of the WT cells was decreased compared with that of the unstressed WT cells. These observations imply that *TIM8* deficiency could also protect against TM in the stationary phase, although not as well as in the growth phase.

### 4.3. Improving the Antioxidant Capacity Further Enhances TM Resistance in the tim8Δ Strain

In the previous section, we showed that the expression levels of most of the investigated antioxidant genes decreased in *tim8Δ* cells under both unstressed and TM-stressed conditions and speculated that the increases in ROS levels and oxidative stress could be a possible reason for the observed ER stress in *tim8Δ* cells. Previous studies have reported that increasing the antioxidant capacity of cells can alleviate ER stress [54,55]. Therefore, we wondered whether improving the antioxidant capacity could relieve ER stress in *tim8Δ* cells.

Considering that the mitochondrial superoxide dismutase-encoding gene, *SOD2*, expression level was the most significantly reduced under unstressed conditions, we used the high copy number vector pAUR123, which has the *ADH1* promoter, to overexpress *SOD2* in *tim8Δ* cells. RT-qPCR confirmed *SOD2* overexpression in these *tim8Δ* cells, and an ROS assay verified that the intracellular ROS level was indeed lower in *tim8Δ SOD2*OX cells (Figure 5B) than in *tim8Δ* cells under both unstressed and TM-stressed conditions, suggesting that oxidative stress was lower in *tim8Δ* cells.

The spot assay and colony-forming unit assay revealed that *SOD2* overexpression obviously enhanced the growth ability of the *tim8Δ* strain on agar plates containing TM (the improvement was not obvious under normal conditions) (Figure 5C,D), and the UPR activity in *tim8Δ SOD2* OX cells was lower than that in *tim8Δ* cells under the TM-stressed condition (Figure 5E).

We also overexpressed another antioxidant gene in *tim8Δ* cells, *CTT1*, which encodes a peroxisomal catalase that can break down H_2_O_2_ [56], and the effect was similar to that of *SOD2* overexpression (Figure 5C,D). These observations suggested that improving the antioxidant capacity indeed further enhanced TM resistance in the *tim8Δ* strain, although it did not obviously enhance the growth ability of or reduce ER stress in *tim8Δ* cells under normal conditions.

Notably, although overexpression of *SOD2* decreased the ROS levels in the *tim8Δ* strain under both unstressed and TM-stressed conditions and enhanced TM resistance, the shortened CLS and impaired mitochondrial respiration capacity of *tim8Δ* cells were not reversed by *SOD2* overexpression (Figure 5G,H). We speculate that this might be due to severe oxidative stress and ER stress in *tim8Δ* cells, which cannot be reversed through improvements in antioxidant capacity caused by the overexpression of one or two antioxidant genes.

Moreover, since *TIM8* deficiency could lead to ER stress in yeast cells under the unstressed condition, we also wondered whether further upregulating the ER stress response could restore CLS in *tim8Δ* cells. We overexpressed the spliced *HAC1* in *tim8Δ* cells. The results showed that the *tim8ΔHAC1*OX cells exhibited a CLS similar to that of *tim8Δ* cells. In addition, the TTC assay results also showed there was no difference between the *tim8ΔHAC1* OX strain and the *tim8Δ* strains. We speculated these observations might be due to the existence of basic UPR activity in *tim8Δ* cells, and a more enhanced ER stress response will not further enhance the CLS of *tim8Δ* cells.

### 4.4. Knockdown of TIMM8A Induces ER Stress

Next, we asked whether the *TIM8* gene function is conserved among its homologs in mammalian cells. Unlike budding yeast, the UPR is initiated in mammals by three distinct classes of ER transmembrane proteins: IRE1, PERK, and ATF6 [8,57].

We used the human RPE cell line ARPE-19 as a model to test our hypothesis. RPE cells are a good model for exploring oxidative stress and ER stress, the latter of which is an important feature of age-related macular degeneration (AMD), the most common cause of irreversible vision loss, especially in elderly individuals [58].

We used RNA interference to silence the TIMM8A gene and found that the protein levels of GRP78 and XBP1s, two widely used markers of ER stress in the literature [59,60], clearly increased. High GRP78 protein levels are dependent on the activation of the IRE1, PERK, and ATF6 UPR pathways. The XBP1s protein (homologous to yeast *HAC1*) is specifically induced by activated IRE-1 under ER stress and upregulates chaperones involved in the restoration of protein folding or the degradation of unfolded proteins in the ER.

Moreover, we found that the intracellular ROS levels were increased in TIMM8A-knockdown cells, similar to budding yeast. Intracellular Ca^2+^ plays a crucial role in stress responses, and the ER is the main organelle that stores Ca^2+^. Abnormal intracellular Ca^2+^ levels are associated with ER stress [61], and disrupted ER Ca^2+^ homeostasis causes the accumulation of misfolded proteins in the ER [62,63]. Therefore, we detected the intracellular Ca^2+^ level and found that it increased in TIMM8A-knockdown cells.

As mentioned above, these observations indicate that TIMM8A knockdown could induce ER stress similar to that induced by the deletion of *TIM8* in yeast cells, suggesting that the function of the *TIM8* gene is conserved in the ER stress response.

## 5. Conclusions

In summary, the results of the present study revealed that yeast *TIM8* deficiency could induce the intracellular ER stress response and chronological senescence, which were accompanied by an increase in the basic UPR. Additionally, we provide evidence that TIMM8A knockdown in human RPE cells can also induce ER stress, highlighting that the potential function of the *TIM8* gene in ER stress is conserved from budding yeast to higher eukaryotes.

## Figures and Tables

**Table 1:** The *S. cerevisiae* strains used in this study.

Strain Name	Genotype	Comments	Source
BY4742	*MATα his3Δ1 leu2Δ0 lys2Δ0 ura3Δ0*	WT	Gift from Matt Kaeberlein
*tim8Δ* *tim8Δ SOD2* OX	BY4742 *tim8::URA3* BY4742 *tim8::URA3SOD2*OX	Deletion of *TIM8* in BY4742 pAUR123*SOD2* was transformed into *tim8Δ*	This study This study
*tim8ΔCTT1* OX *tim8ΔHAC1* OX	BY4742 *tim8::URA3 CTT1* OX BY4742 tim8::URA3*HAC1*OX	pAUR123 *CTT1* was transformed into *tim8Δ* pAUR123 *HAC1* was transformed into *tim8Δ*	This study This study

**Table 2:** The real-time PCR primers used for oxidative stress response assay.

Gene	Primers	Sequence
*PRP8*	Forward	TCATGGCTGCGTCTGAAGTA
	Reverse	GGCACCGTTATTAGCAGCAT
*SOD1*	Forward	AATCCGAGCCAACCACTGTC
	Reverse	CGACGCTTCTGCCTACAACG
*SOD2*	Forward	GCATTACACCAAGCACCAT
	Reverse	CTCGTCCAGACTGCCAAAC
*CTA1*	Forward	CCAACAGGACAGACCCATTC
	Reverse	TTACCCAAAACGCGGTAGAG
*CTT1*	Forward	GATTCCGTTCTACAAGCCAGAC
	Reverse	GGAGTATGGACATCCCAAGTTTC
*GPX1*	Forward	ATCCATTCCCCTTCAACTCC
	Reverse	TCCAGACTTCCCGCTTAC
*GPX2*	Forward	AAAAGCCAAAAAGCAGGTTTACT
	Reverse	CCAAGGACGATGGTTTTGTT
*GPX3*	Forward	TAAAGGGAAAAGTGGTGC
	Reverse	TTCATAATGGGGAAAGTCA
*TRX2*	Forward	AAAGTTTGCAGAACAATATTCTGACG
	Reverse	TTGGCACCGACGACTCTGGTAACC
*MXR1*	Forward	ACAGATTTTGCGGAGGTTTTAC
	Reverse	CCATTTTGGTTGCCATTCTT
*TSA1*	Forward	TCTTTTCGCCTCCACTGACT
	Reverse	CGATGATGAACAAACCTCTCAA
*GLR1*	Forward	CGAACACCAAGCATTACGATTA
	Reverse	GTAGCGAGGTCAGAAGCATACC
*GSH1*	Forward	GACACCGATGTGGAAACTGA
	Reverse	CCCTTTTTGGCATAGGATTG
*GSH2*	Forward	CACAGAGCAGGAAATAGCG
	Reverse	TTGGAGCCAGATAATTGAGT
*YAP1*	Forward	ATGATGTCGTTCCATCTAAGGAAGG
	Reverse	CAACCCCTCTTTCTGAACATTTTGC
*SKN7*	Forward	CCCGAGGAAAGACAGAGATGTA
	Reverse	CAAAAGAGACCCAGAAGGATTG

**Table 3:** The real-time PCR primers used for UPR assay.

Gene	Primers	Sequence
*HACIs*	Forward	GCGTAATCCAGAAGCGCAGT
	Reverse	GTGATGAAGAAATCATTCAATTCAAATG
*EUG1*	Forward	TATCAATCCACTTGCCAAACACTAC
	Reverse	ACCACTGAGTTAGAGCAACGGAA
*ERO1*	Forward	ATGGTGGTAAGCAAGCTGGTC
	Reverse	ACCGATAGAGGCATGGAAACC
*LHS1*	Forward	CCAGGTGAACAGCAGCATTATAT
	Reverse	CTATTGTAACGGGCTGAGTAGTGTC
*KAR2*	Forward	ATACGAGGGTGAAAGAGCCATG
	Reverse	TCGGATTTACCAGTTCCCTTATCT
*FKB2*	Forward	AATCGGGAACTGTATTTGACTCAA
	Reverse	TTGGAATTTGCAGCTTTCTTTT
*INO1*	Forward	TGTTCTGTTGTCGGGTTCCTAAT
	Reverse	CCTTGTACGTGCACTTGTCGGT
*PDI1*	Forward	CATTCCAGGGTTCCCAAGC
	Reverse	CGGATTGGACGATAACTGGAG

## Data Availability

All data used to support the findings of this study are included within the article.

## Supplementary Materials

The following supporting information can be downloaded at: https://www.mdpi.com/article/10.3390/biom15020271/s1, File S1: Raw data of GEL images (and repeats) used in the manuscript. File S2: Raw data of Western blot images (and repeats) used in the manuscript. File S3: RT-qPCR results of the *CTT1* expression levels (in *tim8ΔCTT1*OX cells) and *HAC1* expression levels (in *tim8ΔHAC1*OX cells).File S4: Spot assay results. Original images of Figure 2D and Figure 6A can be found in Supplementary Materials.
