# Supplementary material for: TIM8 Deficiency in Yeast Induces Endoplasmic Reticulum Stress and Shortens the Chronological Lifespan
Source: Biomolecules. 2025 Feb 12;15(2):271. doi: 10.3390/biom15020271 (PMC11853210; doi:10.3390/biom15020271)
Supplement: Supplementary file 1 [file biomolecules-15-00271-s001.zip › biomolecules-3346863-supplementary/File S2.pdf]

Raw data of Western blot images (and repeats) used in the manuscript (Figure 6A).

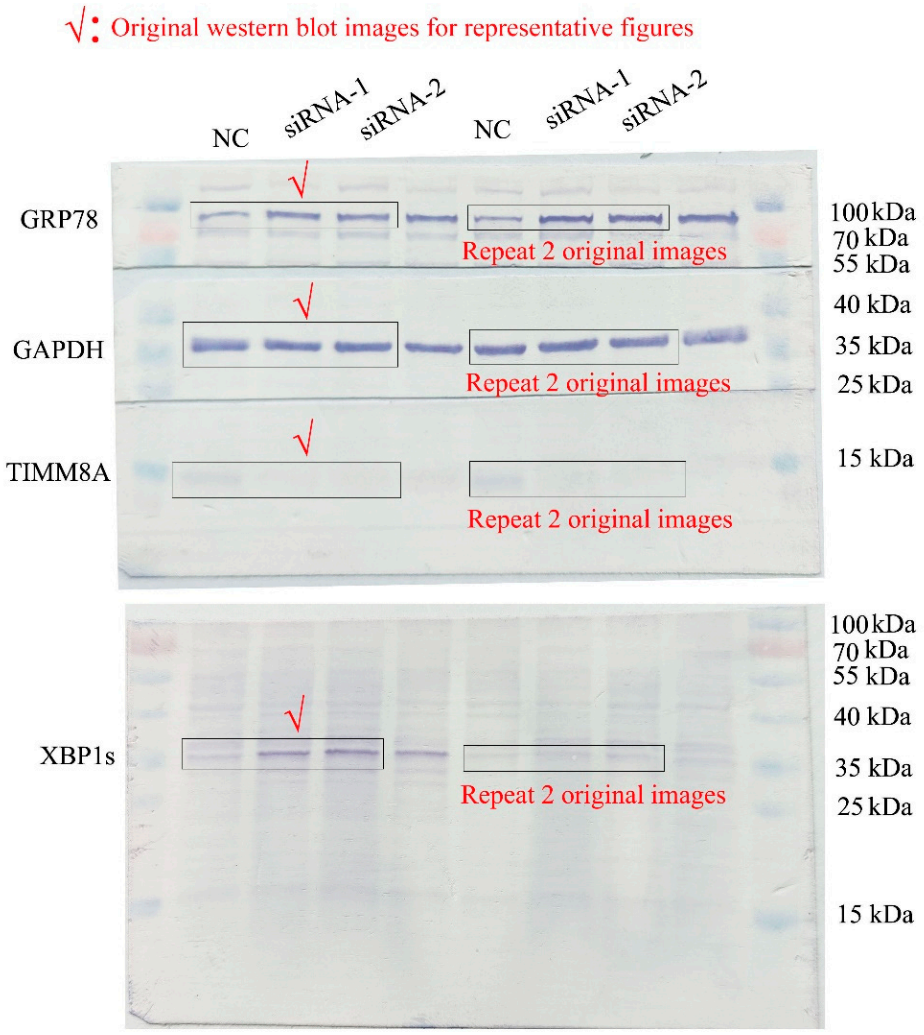

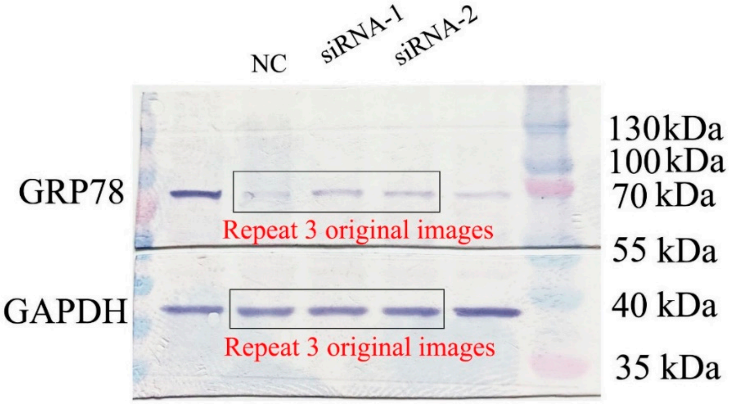
NC
siRNA-1
siRNA-2
GRP78
GAPDH
130kDa
100kDa
70 kDa
55 kDa
40 kDa
35 kDa
Repeat 3 original images
Repeat 3 original images

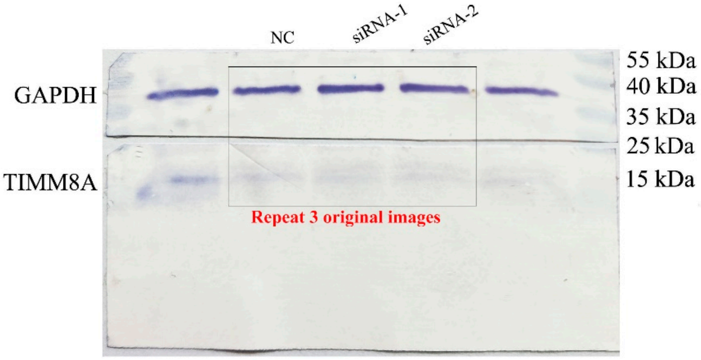
NC
siRNA-1
siRNA-2
GAPDH
TIMM8A
55 kDa
40 kDa
35 kDa
25 kDa
15 kDa
Repeat 3 original images

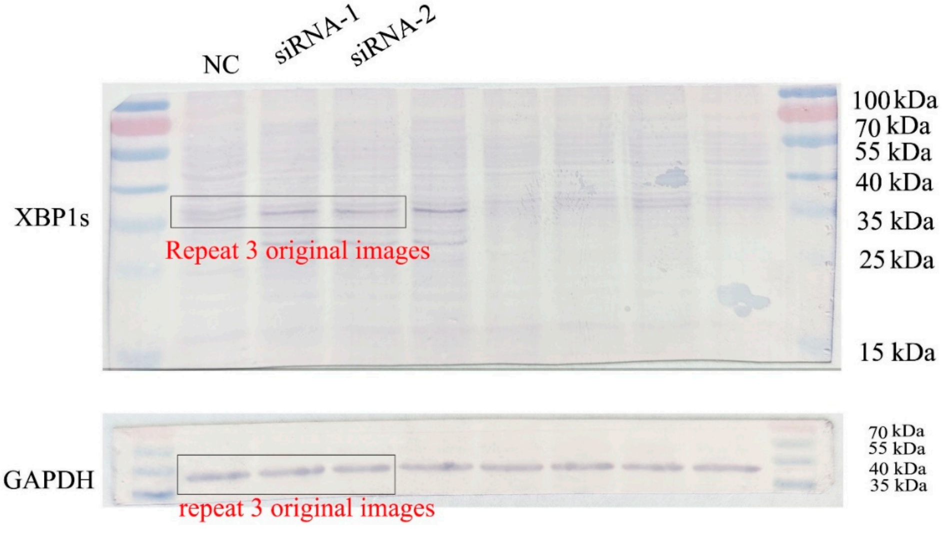
NC
siRNA-1
siRNA-2
XBP1s
GAPDH
100kDa
70 kDa
55 kDa
40 kDa
35 kDa
25 kDa
15 kDa
Repeat 3 original images
70 kDa
55 kDa
40 kDa
35 kDa
repeat 3 original images

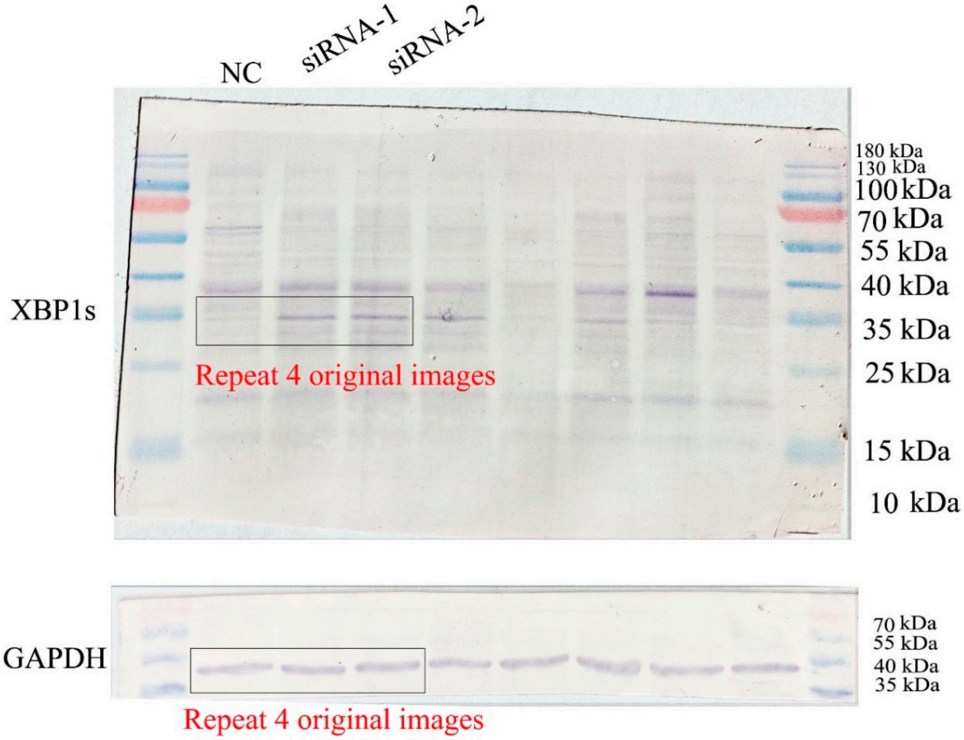
NC
siRNA-1
siRNA-2
XBP1s
Repeat 4 original images
180 kDa
130 kDa
100 kDa
70 kDa
55 kDa
40 kDa
35 kDa
25 kDa
15 kDa
10 kDa
GAPDH
70 kDa
55 kDa
40 kDa
35 kDa
Repeat 4 original images
