# Supplementary material for: TIM8 Deficiency in Yeast Induces Endoplasmic Reticulum Stress and Shortens the Chronological Lifespan
Source: Biomolecules. 2025 Feb 12;15(2):271. doi: 10.3390/biom15020271 (PMC11853210; doi:10.3390/biom15020271)
Supplement: Supplementary file 1 [file biomolecules-15-00271-s001.zip › biomolecules-3346863-supplementary/File S3.pdf]

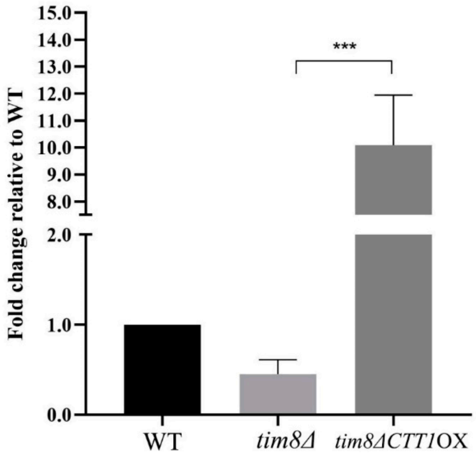

Fig.1 RT–qPCR demonstrated that *CTT1* was overexpressed in *tim8ΔCTT1*OX cells.

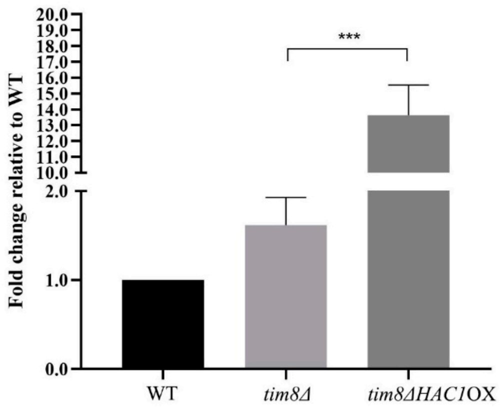

Fig.2 RT–qPCR demonstrated that *HAC1* was overexpressed in *tim8ΔHAC1*OX cells.
