# Supplementary material for: TIM8 Deficiency in Yeast Induces Endoplasmic Reticulum Stress and Shortens the Chronological Lifespan
Source: Biomolecules. 2025 Feb 12;15(2):271. doi: 10.3390/biom15020271 (PMC11853210; doi:10.3390/biom15020271)
Supplement: Supplementary file 1 [file biomolecules-15-00271-s001.zip › biomolecules-3346863-supplementary/File S4.pdf]

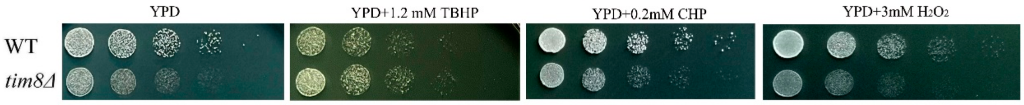

The WT and *tim8Δ* strains were serially diluted and spotted on YPD plates containing 1.5 mM TBHP, 0.2mM CHP or 3mM H_2O_2. The plates were incubated at 30°C until colonies formed, after which photographs were taken.
