# Supplementary figures and images for: TIM8 Deficiency in Yeast Induces Endoplasmic Reticulum Stress and Shortens the Chronological Lifespan
Source: Biomolecules. 2025 Feb 12;15(2):271. doi: 10.3390/biom15020271 (PMC11853210; doi:10.3390/biom15020271)

Raw data of GEL images (and repeats) used in the manuscript (Figure 2D).

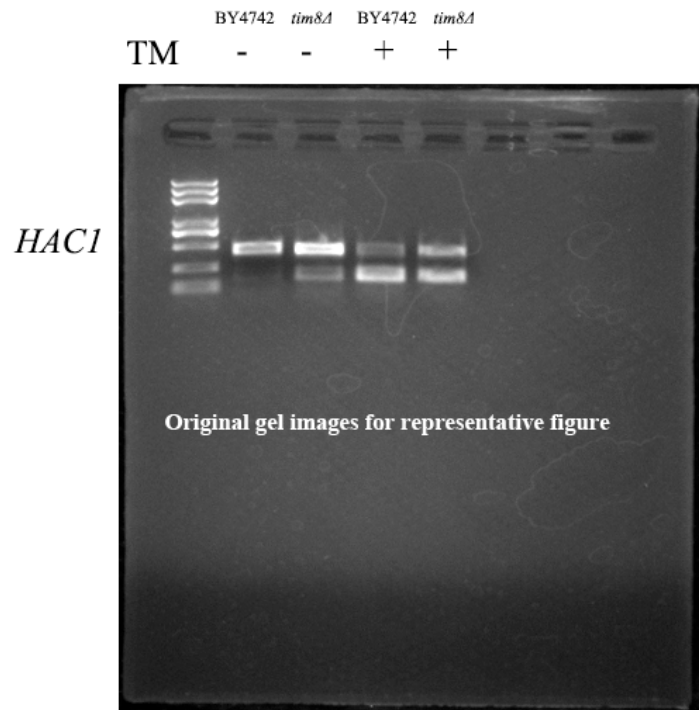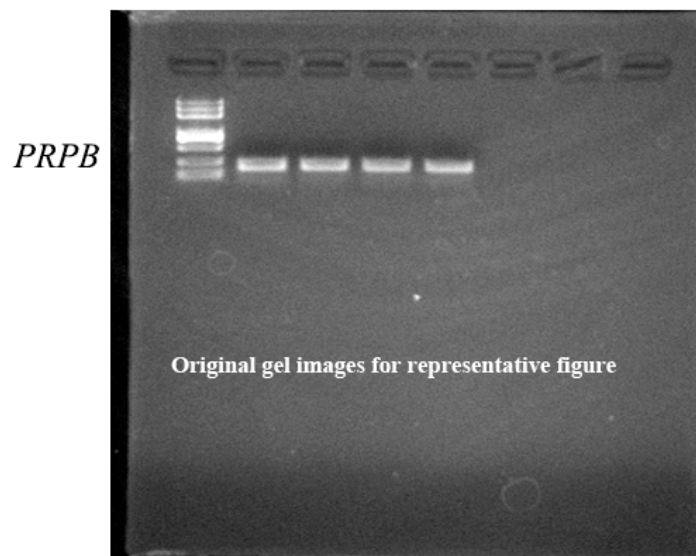

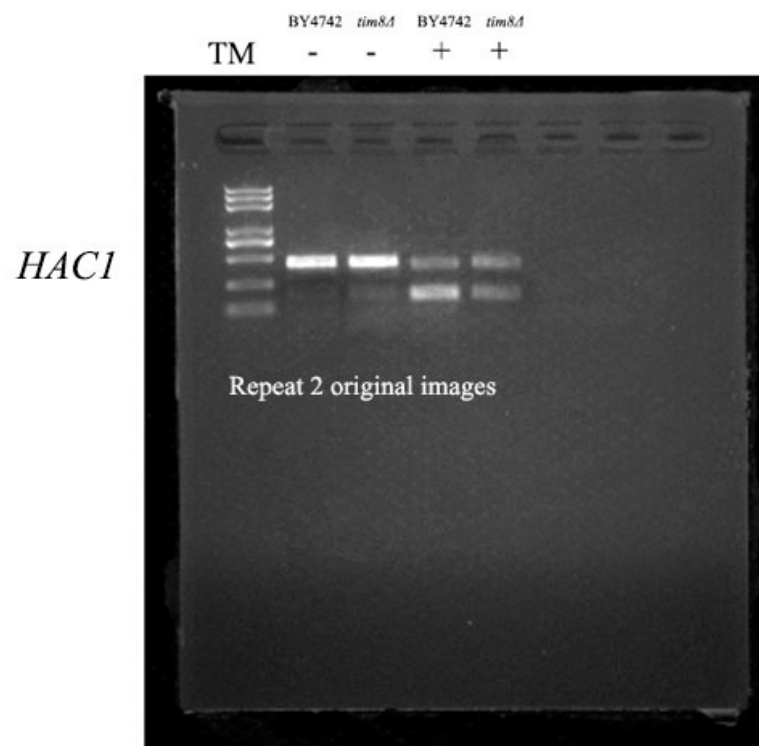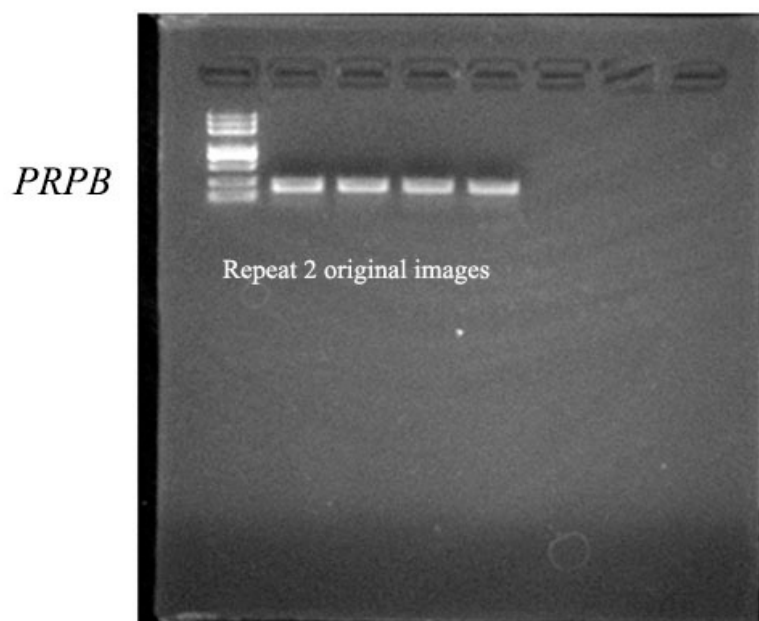

|    |        |              |        |              |
|----|--------|--------------|--------|--------------|
|    | BY4742 | <i>tim8Δ</i> | BY4742 | <i>tim8Δ</i> |
| TM | -      | -            | +      | +            |

*HAC1*

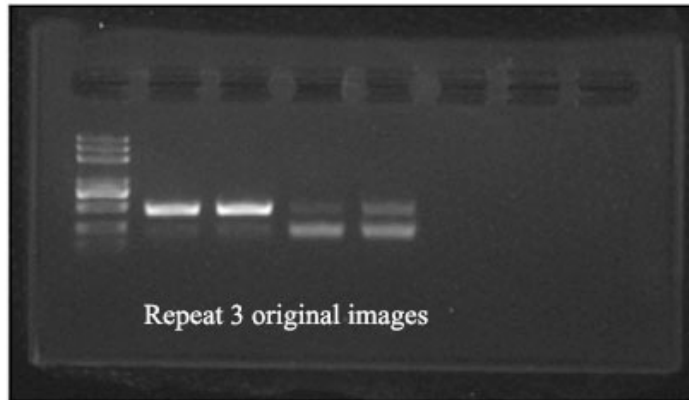

*PRP8*

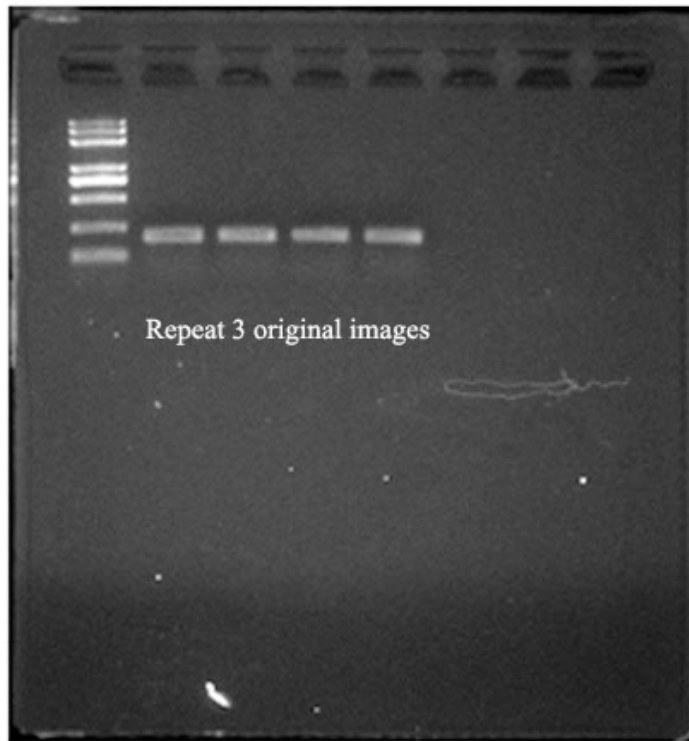

Supplement: Supplementary file 1 [file biomolecules-15-00271-s001.zip › biomolecules-3346863-supplementary/File S1.pdf]
